# Activity Pattern and Correlation between Bat and Insect Abundance at Wind Turbines in South Sweden

**DOI:** 10.3390/ani11113269

**Published:** 2021-11-15

**Authors:** Johnny de Jong, Lara Millon, Olle Håstad, Jonas Victorsson

**Affiliations:** 1 Swedish Biodiversity Centre (CBM), Department of Urban and Rural Development, Swedish University of Agricultural Sciences, Box 7012, 75007 Uppsala, Sweden; 2 Calluna AB, Linköpings Slott, 582 28 Linköping, Sweden; lara.millon@calluna.se; 3 Faculty of Veterinary Medicine and Animal Science, Swedish University of Agricultural Sciences, Box 7084, 750 07 Uppsala, Sweden; olle.hastad@slu.se; 4 Kalmar County Administration, Regeringsgatan 1, 39231 Kalmar, Sweden; jonas.victorsson@lansstyrelsen.se

**Keywords:** wind-power, bat activity, Chiroptera, insect abundance, stop-regulation

## Abstract

**Simple Summary:**

Wind power is an important energy system in the global transition towards renewable energy. As new wind farms are erected in increasing numbers, they will have an impact on many organisms, e.g., through habitat changes and collision mortalities. In this study, we measure bat activity, insect abundance, and weather conditions to test the hypothesis that insect abundance attracts bats to wind turbines because of feeding opportunities. We found that the relationship between insect abundance and bat activity was relatively weak, providing some support for the feeding-attraction hypothesis. However, we also found a strong correlation between bat passes and weather conditions. This suggests that stop-regulation based on weather conditions might be a solution to avoid collisions. However, this study highlights some of the problems with defining the limits for stop-regulation, as bat activity may be high also at relatively high wind speeds and low temperatures.

**Abstract:**

We present data on species composition and activity of bats during two years at three different wind- turbines, located in south Sweden, both at the base and nacelle height. To test the hypothesis that bats are attracted to wind turbines because of feeding opportunities, insects were sampled at nacelle height at one wind turbine using a suction trap, simultaneously as bat activity were measured. At this wind turbine, we also compared two different technical systems for ultrasound recordings and collect meteorological data. The variation in bat activity was high between nights and between wind turbines. In addition to the expected open-air foraging species (*Pipistrellus*, *Nyctalus*, *Vespertilio* and *Eptesicus*), some individuals of unexpected species (*Myotis*, *Barbastella*, and *Plecotus*) were found at nacelle height. There was a weak but significant positive relation between bat activity and insect abundance, so the hypothesis could not be rejected, suggesting there might be other factors than insect abundance explaining the frequency of bat visits at the nacelle. We found a strong correlation between bat passes and weather conditions. A reasonable way to mitigate collisions is with stop-regulation. However, this study highlights some of the problems with defining the limits for stop-regulation based on weather conditions.

## 1. Introduction

Wind power is increasing in importance as future energy systems and the construction of a large number of new wind farms are being planned [1]. This expansion will cause an increased impact on wildlife and efficient mitigation methods are therefore urgently needed [2]. Bats are among the organisms heavily affected, not only due to high mortality caused by collisions [3], but also because of barrier effects and habitat loss [4,5,6]. A number of hypotheses explaining the behaviour of bats and associated fatalities at wind parks have been suggested in the literature. These include passive factors such as the wind farm creating physical barriers to migrating bats, and active factors such as attraction to the wind turbines for foraging, roosting, or mating [3,7,8,9]. There is some support for several of the hypotheses and most probably, the explanation is a combination of several factors [9]. In this study, we focus on the feeding-attraction hypothesis, which states that the occurrence of bats at the turbine nacelle is due to their attraction to relatively high insect abundances for foraging [7]. There might be several reasons for insects to concentrate around the nacelle, such as light and colour [10], but also the physical structure may attract insects. For example, flies (order Diptera) have a swarming behaviour in which individuals of both sexes concentrate in groups close to marked objects in the landscape [11]. The swarm may become large, and under good conditions, contain thousands of individuals. During typical mosquito swarming behaviour, the individuals in the swarm turn towards the wind causing it to remain stable above, or in the shelter behind, a marked upright object [11]. This behaviour can lead to accumulations of insects above or behind the nacelle. Another reason for high insect abundance at nacelle-height is that during migration, insects use high-altitude movements [12,13] aided by wind streams to colonize new sites. Insects actively move vertically to find heights with appropriate wind directions [14]. 

Rydell et al. [15] discussed the feeding-attraction hypothesis and found it most likely that bat occurrence at nacelle height was linked to nocturnal insect migration. Several studies have also confirmed that bats are actively foraging at wind turbines [9,16,17,18]. The feeding-attraction hypothesis was tested in the field by Foo et al. [18], who measured insect and bat presence at wind turbines in North Texas, United States. They compared the stomach contents of bats with the contents of bat faeces and demonstrated that bats were foraging and resting at the nacelle. On the other hand, Reimer et al. [19] found no support for active foraging when studying two migrating bat species. In their study, they compared feeding activities at different heights, and between wind turbines and meteorological towers, but found no differences in feeding activity. However, Rydell et al. [20] showed that bats sometimes fed on diurnal flies resting on wind turbines and wind towers and argued that the absence of feeding buzz does not exclude the possibility that the bats are feeding. 

Measuring bat activity at nacelle height is difficult for several reasons. Visiting the tower requires special education, and during the work, the turbines must be stopped. However, several studies of bats at nacelle height have been made in connection to environmental impact assessment [21,22,23]. Unfortunately, there are just a few such studies published in scientific papers. In Canada, Baerwald and Barclay [24] and Reimer et al. [19] studied the activity pattern at 67 m height. In the former study, the mean activity of five migratory bat species was described. In France, Roemer et al. [25] investigated bat activity at 50–100 m above ground and suggested that their results may serve as a reference for the flight behaviour and time spent at different heights. However, both species composition and bat abundance differ considerably between Scandinavia and other parts of Europe and North America, and these results may not be applicable elsewhere [22].

In order to mitigate bat fatalities at wind farms, it is important to understand the mechanisms behind the bat occurrence at nacelle height. Several mitigation methods have been proposed to avoid bat collisions, such as operational adjustments [26,27,28,29,30]. In Sweden, the recommendation is to turn the wind turbines off in late summer and autumn at temperatures above 14 °C and wind speeds below 6 m/s [22]. This recommendation is based on a literature review (based on the activity of bats at six wind turbines), which showed that most collisions occur during August through September, at relatively high temperatures and at low wind speeds. The proposal makes sense, if the explanation for the bats’ presence at the wind turbines is the weather. Thus, it is important to have good knowledge of the factors affecting insects, bat abundance, and the variation of both within and between years. 

The objectives of this study were to: (a) test the feeding attraction hypothesis by measuring bat activity and insect abundance at nacelle height, and compare the effect of the insect abundance and weather conditions on bat activity (b) study the variation of species composition and bat activity in time (between nights and between years) and space (ground level and nacelle-height, and between sites), (c) compare two technical systems for bat recordings at nacelle-height, (d) based on the result, discuss stop-regulation as a potential mitigation method.

We present data on bat activity at three wind turbines between June and October 2017 and 2018 at nacelle height and at the ground. Data on insect abundance were collected at nacelle-height for one year at one wind turbine, with a new type of suction trap, which made it possible to measure insect abundance at the same time as bats were active. Meteorological data were collected at nacelle-height at the three wind turbines.

## 2. Materials and Methods

The data were collected June–October 2017 and 2018 at three wind turbines (Stena Renewable AB, Göteborg, Sweden and Erikhester vindpark AB, Vetlanda, Sweden), located at two different sites in the province of Småland in southern Sweden (south of the city Vetlanda, 57°25′38″, 15°05′07″). The three wind turbines were part of bigger wind farms (with 6 and 32 turbines) and were selected based on practical reasons in cooperation with the company. The surrounding landscape was dominated by managed coniferous forests with no large water bodies such as lakes or coastline nearby. At the southern site, bat activity was measured in 2017 and 2018 at one wind turbine (hereafter called southern wind turbine, SWT), both at the base of the wind turbine (about 5 m above the ground), and at nacelle height (about 130 m above the ground). At the northern site, the bat activity was only measured at one wind turbine in 2017 and at two wind turbines in 2018 (hereafter called the northern wind turbines, NWT1 and NWT2) at nacelle height (about 150 m above the ground). The distance between SWT and NWT1 was 13 km, and the distance between NWT1 and NWT2 was 1.6 km. 

At all sites, wind speed (m/s) and temperature (°C) were measured at the nacelle height every 10 min (data from the wind-power companies). Average wind speed and average temperature were calculated per night and per wind turbine between sunset and sunrise times. Precipitation (mm) was measured at Målilla weather station every hour (weather station of the Swedish meteorological and hydrological institute), which is within 50 km of each wind farm. Total precipitation was calculated per night between sunset and sunrise.

Bat activity was measured by using ultrasound detector Avisoft 116 hnbm attached to the microphone EP3 (Bioacoustic technology Gmbh, Winkelhaid, Germany). The recordings took place every night during the study period from June until October. At the SWT location, we deployed both Avisoft 116 hnbm and Pettersson D500X detectors (Pettersson Elektronik AB, Uppsala, Sweden) with microphones placed next to each other, to estimate the impact of different systems on the results. The settings of both ultrasound detectors are presented in Appendix A. The Pettersson system is commonly used in Sweden [22], while Avisoft is more commonly used throughout parts of Europe [31]. Unfortunately, the Avisoft recorder at the nacelle height at the SWT did not work for a period in 2018. Thus, we did not include data collected from the Avisoft recorder at nacelle height, at the SWT in the analysis.

Sorting and preliminary sound analysis was conducted using the software program Omnibat, which provided an automatic identification (Developed by Johan Karlsson & Alexander Eriksson at Ecocom AB, Kalmar, Sweden, but not available on the market). All files containing bat calls were then investigated manually with Omnibat and Batsound 4.03 (Pettersson Elektronik) to identify the species. Species within the genus *Myotis* are difficult to separate and were combined into *Myotis* spp. The most difficult pulses were assigned to microchiroptera group. Each recording containing a bat call was counted as one bat pass. If there were several sounds of the same species in one recording, it was counted as one bat pass. However, several species in one recording were counted as separate bat passes. Bat activity (total bat activity and bat activity per species) was calculated for each device (D500X or Avisoft), each height (base or nacelle height) and each night (Table 1). 

At SWT, insect abundance was measured at the nacelle height during 2017, between the 28th of June and the 31st of October. However, during a 21-day period, the trap was active all day and all night instead of all night only. This was an intentional change to verify the functionality of the trap. The data from this 21-day period were excluded from the analyses. Insects were trapped by using a suction trap placed close to the nacelle at SWT. Suction traps [32] are often used for the estimation of food availability for bats [33,34,35]. It works as a vacuum cleaner in which the insects are separated from the airflow by a net and then collected in a bottle with glycol. To enable collection of separate samples for each night, the trap contained a revolver magazine of 21 bottles. The trap was programmed to change bottles every day. The fan was synchronised to start at sunset and stop at sunrise. A camera was placed within the trap, sending pictures every day to make it possible to verify the functionality of the device. Every third week the bottles were replaced. The fan had a diameter of 50 cm, creating an airflow of 6000 m^3^/hour through the trap. Insects were identified to order, and when possible, to sub-order and counted, thereby providing an abundance measurement for each night. 

Statistical analyses were performed with SAS proc GENMOD, version 9.4 (SAS, Cary, NC), and R, version 4.0.4 (R foundation for statistical computing, 2021).

The difference in wind speed and temperature between 2017 and 2018 were controlled with the Student’s *t*-test. Pearson rank correlation was used to calculate the correlation between the wind and the temperature. Only the weather conditions from SWT and NWT1 were used for those statistics.

A multiple Poisson-regression model was used to explain the relationship between the insect abundance and the three weather variables (as insect abundance, the response variable, was not normally distributed, [36]). The three weather variables (temperature, wind, precipitation) were used as explanatory variables. 

The relationship between bat activity, insect abundance, and weather (wind, temperature and precipitation) from SWT were also analysed using multiple Poisson regressions, because data on bat activity were not normally distributed [36]. Insect abundance and the three weather variables were used as explanatory variables to analyse the number of bat-passes per night for the total bat activity or the activity of each of the five most common taxa. There was no risk of multicollinearity [36], because the correlations between the explanatory variables varied between −0.32 and + 0.08. All models containing all possible combinations of the four explanatory variables were analysed (in our case 14 models). Interactions were not included. Among these 14 models, the one with the lowest AIC (Akaike information criterion), in combination with *p* < 0.2 for all included variables, was chosen. Dscale in proc GENMOD was used to compensate for the overdispersion of the data when needed [37]. However, the overdispersion of the data with the activity of *E. nilssonii* as the response variable could not be compensated. Therefore, the models with *E. nilssonii* activity were done without the outlier (where the overdispersion was compensated). In addition, the model with total bat activity was tested without the same outlier. 

The relations between bat activity and weather conditions were also examined at NWT. A generalized linear mixed model was used, with the total bat activity as the response variable, and the three meteorological variables as explanatory variables. The date was used as a random effect. The correlation between the wind speed and the temperature was low (−0.29), so these two variables could be used in the same model [36].

In order to know if bat activity differed between 2017 and 2018, Wilcoxon tests were used to compare bat activity at nacelle height at NWT1 and to compare bat activity at SWT at ground height (data from the Avisoft equipment). The comparisons were made for the total bat activity and for each of the five most common species.

In the same way, bat activity between two wind turbines was compared with the Wilcoxon test. In 2017, bat activity was compared between SWT and NWT1 and in 2018 between NWT1 and NWT2 (data from the Avisoft equipment). 

We compared a number of recordings and the correlation between two technical systems (D500X and Avisoft) at the ground level at SWT in 2018 by using the Wilcox test for paired data and Spearman rank correlation.

## 3. Results

In total, recordings were collected at nacelle height 243 and 403 nights in 2017 and 2018 respectively, and at ground level 91 and 233 nights in 2017 and 2018 respectively (Table 1). Nine taxa were identified, and more than 7 000 bat passes were recorded (Table 1). The most common species were *Pipistrellus pygmaeus* (32% of the total number of bat passes), *Eptesicus nilssonii* (29%), *Myotis* spp. (20%), *Nyctalus noctula* (10%). There were also a few records of *Vespertilio murinus* (4%) and *Eptesicus serotinus*, *Barbastella barbastellus*, *Pipistrellus nathusii* and *Plecotus auritus* (<0.01% each). All species except *P. nathusii* were present at the nacelle height, and all species except *B. barbastellus* were present at the ground level. *E. nilssonii*, *P. pygmaeus* and *Myotis* spp. represent the three most common taxa at nacelle height and at ground level. 

Weather conditions during the survey period are shown in Table 2. There was no significant difference in wind speed between 2017 and 2018 (t = −0.32, *p*-value = 0.74, n = 306 nights). However, the mean temperature was significantly lower in 2017 compared to 2018 (t = −6.38, *p*-value < 0.01, n = 306 nights). Temperature decreased with an increasing wind-speed (data from 2017 and 2018, Pearson rank correlation r_s_ = –0.30, *p* < 0.01, n = 306 nights). In 2017, there were 34 nights of rain and 11 in 2018. Precipitation varied between 0.1 mm and 20.0 mm of rain in 2017 and between 0.1 mm and 21.4 mm in 2018 during rainy days. 

**Figure 1:**
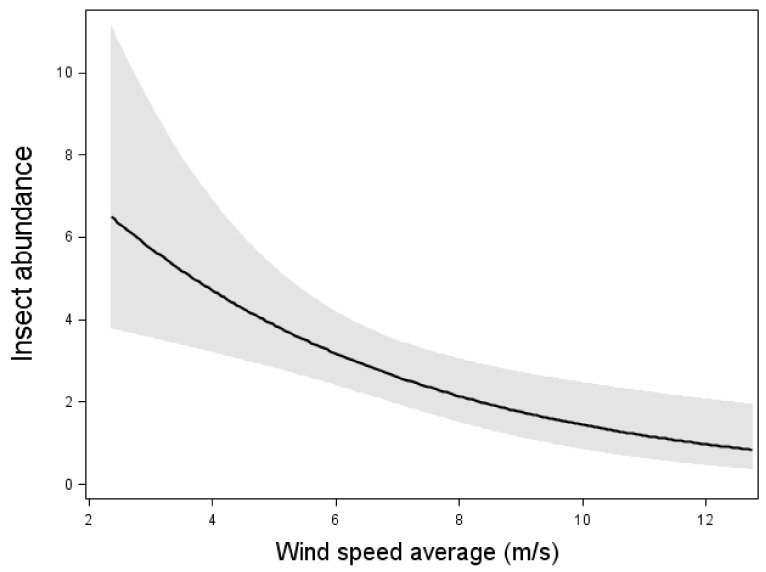
Relation between insect abundance and mean wind speed. Results from multiple Poissonregression, where the insect abundance was the response variable. Relation, test-variable and *p*-value are shown for wind speed, which was the only significant variable in the model with the lowest AIC value. The line shows the mean value and dark area shows 95% confidence interval. n = 105 nights.

The model including insect abundance as the response variable and weather conditions as explanatory variable showed that there was a negative relation between wind speed and insect abundance (Wald χ^2^ = 10.18, *p* < 0.01, n = 105 nights, Figure 1). However, the temperature did not affect insect abundance at the nacelle level.

Bat activity, insect abundance and weather conditions were measured simultaneously during 87 nights in 2017 at SWT at nacelle height. Table 3 summarizes the results for all selected models, including bat activity, insect abundance, and weather conditions at SWT.

**Figure 2:**
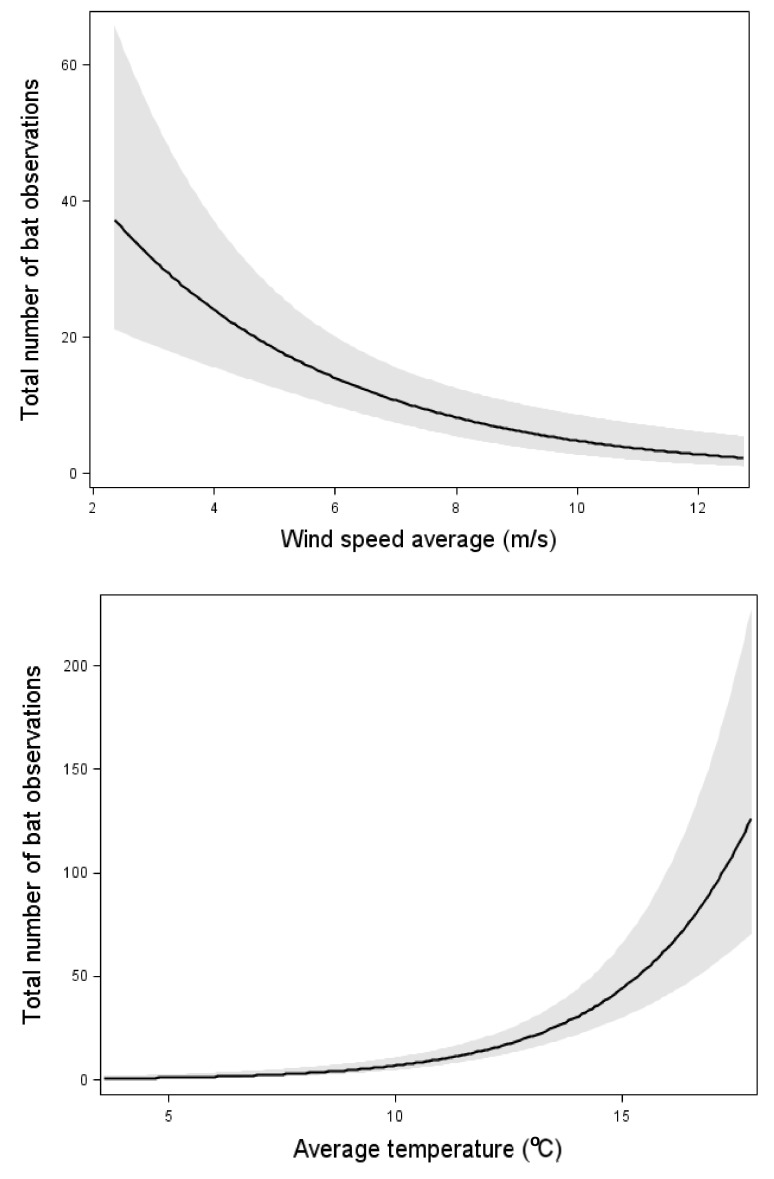

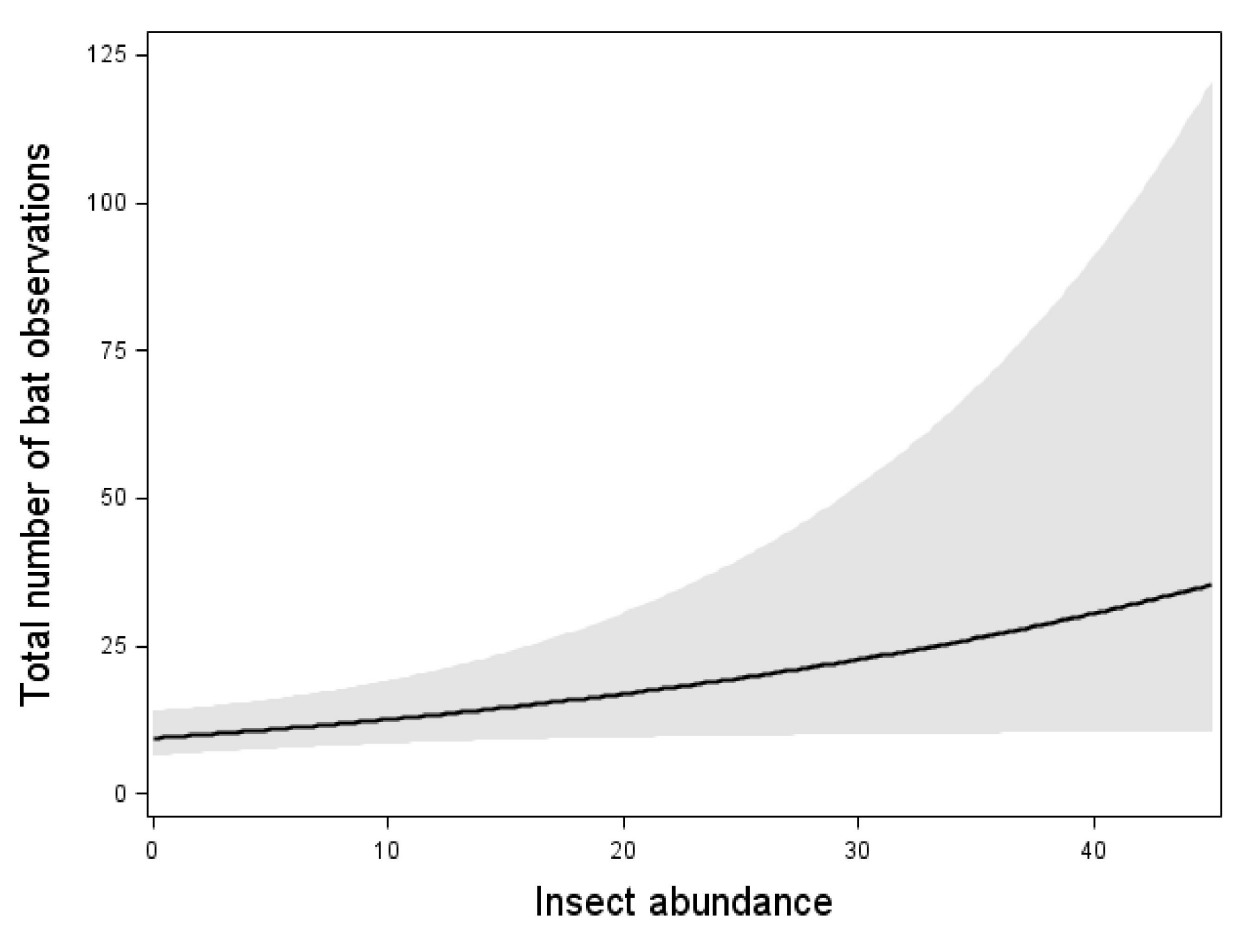
Relation between the total bat activity and the wind speed, temperature, and insect abundance. Results from multiple Poisson regression, where the total bat activity was the response variable. Relation, test-variable, and *p*-value are shown for all variables included in the model with the lowest AIC value (Table 3). The line shows the mean value for each explaining variable when the values for all the other variables in the model are kept constant at their mean value. The dark area shows 95% confidence interval. n = 87 nights.

The total bat activity increased with an increasing insect abundance (Figure 2). However, the relation was weaker than the effect of wind speed and temperature (Figure 2, Table 3). When one outlier with the highest bat activity was removed, the significant correlation between insect abundance and total bat activity disappeared. However, the effects of wind speed and temperature remained.

*N. noctula*, *E. nilssonii*, and *V. murinus* all increased their activity with increasing insect abundance (Table 3). Each of these three species were also affected by one of the weather factors: *E. nilssonii* and *V. murinus* increased their activity with increasing temperature, whereas *N. noctula* activity decreased with increasing wind speed (Table 3). The other two taxa, *P. pygmaeus* and *Myotis* spp., that was tested were unaffected by insect abundance but were affected by weather factors. Both *P. pygmaeus* and *Myotis* spp. activity decreased with increasing wind speed and *P. pygmaeus* was also affected by temperature and increased its activity with increasing temperature (Table 3).

With both models with and without data from 11 August, the total bat activity increased with increasing temperature and decreased with increasing wind-speed (Figure 2, Table 3). However, the variation between nights was large and no obvious threshold of bat activity was apparent. Bats were observed up to a wind speed of about 12 m/s, but in the three occasions with higher wind speeds, no observations were made. The same relations between total bat activity and weather conditions were found from the data from NWT: bat activity significantly decreased with an increasing wind speed (estimate: −0.86, *p*-value < 0.01) and significantly increased with an increasing temperature (estimate: 0.28123, *p*-value < 0.01, n = 421 nights). The precipitation had no significant effect on bat activity.

Total bat activity at NWT1 at nacelle height did not significantly differ between 2017 and 2018 (0.43 ± 0.14 bat passes per night in 2017 versus 2.64 ± 1.23 bat passes per night in 2018, Wilcox test = 8997, *p*-value = 0.28, n = 137 nights). The activity of the four main species at NWT1 (*E. nilssonii*, *P. pygmaeus*, *V. murinus* and *N. noctula)* did not differ between 2017 and 2018 at nacelle height (Table 4). Total bat activity at SWT at ground height significantly differed between 2017 and 2018 (2.88 ± 0.80 bat passes per night in 2017 versus 16.93 ± 3.25 bat passes per night in 2018, Wilcox test = 2015.5, *p*-value < 0.01, n = 91 nights). The activity of *E. nilssonii*, *P. pygmaeus*, *Myotis* ssp and *N. noctula* did also significantly differ between 2017 and 2018 at the ground height at SWT. However, there was no significant difference in *V. murinus* activity between 2017 and 2018 at the ground level at SWT (Table 4).

**Figure 3:**
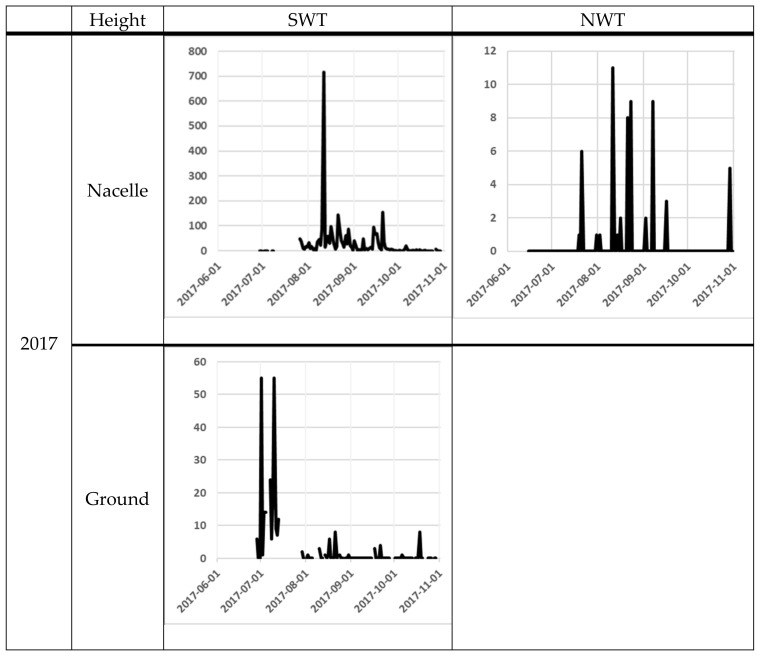

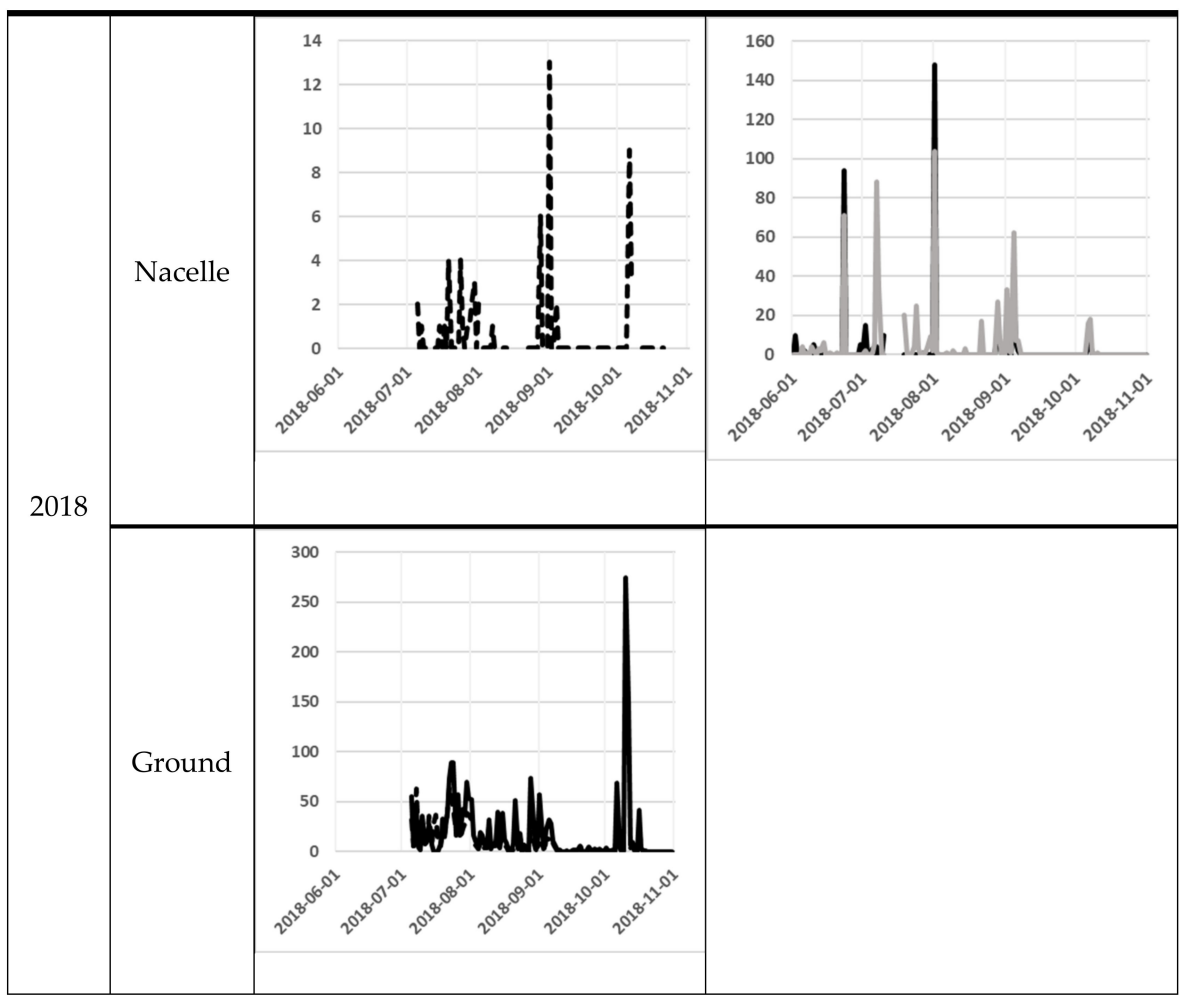
Bat activity per night (*y*-axis) for each night (*x*-axis). The filled line are for the Avisoft data and the dotted line for the D500X data. For the NWT site, the black line is for the NWT 1 and the grey line is for the NWT 2. The scale on the *y*-axis differs between the diagrams, because of the high variation in bat registrations between sites.

The number of bat passes at nacelle level varied significantly from day to day. One of the most obvious differences was between the 11th and the 12th of August at SWT in 2017, when the number of bat passes varied from 717 to 13 the next night. At ground level, the variation was lower, but from the 9th to the 10th October in 2018, bat activity varied between 1 and 275 bat passes (Figure 3). 

At the nacelle level, high bat activity (more than 50 bat passes per night) occurred on several occasions (Figure 3). The earliest peak of activity at the nacelle level was observed at the end of June 2018 at the NWT1 and NWT2 and the latest at the end of September 2017 at SWT (Figure 3). At ground level, the earliest peak of activity was observed at the beginning of July in both 2017 and 2018 at SWT site (note that the recording did not start a long time before the peak of activity). The latest peak of activity at ground level was recorded in mid-October 2018 at the SWT site (Figure 1). 

At SWT site, bat activity was registered at the same time at ground level and nacelle level during 85 nights with Avisoft recorder in 2017 and during 95 nights with D500X in 2018. The number of bat-passes at ground level was not correlated to the number of bat-passes at the nacelle from the dataset from Avisoft from 2017 (Spearman’s rank correlation r_s_ = –0.008, p = 0.94, n = 85 nights). However, when using the dataset from D500X from 2018, there was a significant correlation between bat activity at ground level and nacelle level (Spearman´s rank correlation r_s_ = –0.44, *p* < 0.01, n = 95 nights).

Bat activity differed significantly at the nacelle height in 2017 between SWT and NWT1 (with 26.1 ± 7.3 bat passes per night at SWT and 0.5 ± 0.2 bat passes at NWT1, Wilcox test = 9279.5, *p*-value < 0.01, n = 104 nights). When analysed species by species, the activity of the five main species was also significantly different between SWT and NWT1 at the nacelle height in 2017 (Table 5). Bat activity at NWT site also differed significantly between NWT1 and NWT2 at nacelle height in 2018 (2.6 ± 1.3 bat passes per night at NWT1 and 4.4 ± 1.3 bat passes per night at NWT2, Wilcox test = 10764, *p*-value < 0.01, n = 137 nights). The activity of *E. nilssonii*, *V. murinus* and *N. noctula* also significantly differed between NWT1 and NWT2 at nacelle height in 2018 (Table 5). However, the activity of *P. pygmaeus* did not differ significantly between NWT1 and NWT2 at nacelle height in 2018 (Table 5).

We compared acoustic data recorded using the D500X and Avisoft units at the SWT site in 2018 (total number of nights when both systems worked = 114). The Pettersson unit recorded a higher number of total acoustic files (including noise files). However, the Avisoft unit recorded a higher total number of files with bats (Table 1) and recorded a significantly higher mean nightly bat activity (14.8 ± 2.0 bats per night) than the D500X (and 9.6 ± 1.3 bats per night) (Wilcox test for paired data: V = 3201, *p*-value <0.01, n = 114 nights). The overall pattern of bat activity per night was similar across detectors and the number of bat passes recorded per night were strongly correlated between detectors (Figure 3, Spearman rank correlation r_s_ =−0.83, *p* < 0.01, n = 114 nights).

## 4. Discussion

The importance of wind power as a renewable energy source is increasing, and the impact on bat populations is an urgent problem. Most probably, it will cause population decline for several species, and we need to improve our mitigation efforts considerably [38]. However, this also requires a better understanding of bat biology.

This study shows there was a correlation between insect abundance and bat activity at the nacelle height of a wind turbine. To our knowledge, this is one of the first studies that simultaneously measured insect abundance and bat activity at the nacelle height during the whole period (July until October) when bats aggregate at the nacelle. In addition, it is the first study using a suction trap at the nacelle. Other recent studies also worked on the interaction between bats and insects at wind turbines [9,18,20,39,40]. However, Foo et al. [18] and Ahlén et al. [39,40] did not trap insects at the nacelle height, and Rydell et al. [20] did not measure bat activity at the nacelle level simultaneously with insect collection. In addition, insects were trapped day and night with glue traps instead of just when bats were active. In the study by Jansson et al. [9], insect abundance was measured using a laser beam at nacelle height, at the same time as bat activity was measured with an ultrasound detector from the ground. This was done during only a few nights, but still, the result is interesting since they demonstrated how swarms of insects occurred in the evening, and on some occasions, remained after sunset. 

In our study, insect abundance at nacelle height was low (compared to the ground, own observations), which is similar to the result of Rydell et al. [20]. The effect of insect abundance on bat activity was lower than the effects of temperature and wind speed. One possible explanation is that the different taxa were affected differently by insect abundance—*N. noctula*, *E. nilssonii*, and *V. murinus* all increased their activity with increasing insect abundance, whereas *P. pygmaeus* and *Myotis* spp. were unaffected. Consequently, when we combine the bat observations for all species, the effect of insect abundance is driven primarily by the most common taxa, i.e., *E. nilssonii*, *P. pygmaeus* and *Myotis* spp. and for two of those taxa, there was no effect of insect abundance. An alternative explanation for bat occurrence at the nacelle level is that low wind speed and high-temperature trigger bat movements, and during these periods, bats may be more likely to explore near the nacelle area [24]. This means that favourable weather conditions are a stronger predictor of the presence of bats close to the nacelle of wind turbines than is the presence of insects. If this is the case, measures to decrease insect abundance at the wind turbines, e.g., insect aversive colours, will not have any impact on bat activity [10].

Measuring insect abundance and bat occurrence at nacelle height is associated with several methodological problems [41]. The insect trap measures insects at only one location on top of the nacelle and it is difficult to determine the optimal place to monitor insect abundance. The ultrasound detectors only measures bat passes, without providing information on the number of individuals. Further, neither the position of the flying bat, nor the display of risky behaviour can be deduced from the recordings. Bats may fly close to the nacelle or far away. *N. noctula* and *E. nilssonii* may be recorded when flying more than 100 m away (own observations) [42]. There are also technical limitations. On the one hand, the Avisoft system was much more efficient (given the settings we used) than the D500X. On the other hand, it did not work for some periods and required some support, which might be difficult at a wind farm. 

We found a surprisingly high number of species at nacelle-height including *B. barbastellus*, *P. auritus* and *Myotis* spp. Most often, only open-air foragers are considered as having a high risk for collision [43]. This is mainly based on carcass surveys [44]. It is possible that low-risk species very seldom visit the nacelle area, or are using a different foraging strategy, which makes them less vulnerable [42]. The fact that bat abundance at ground level and bat abundance at nacelle height do not always correlate makes it difficult to estimate the risks for bat fatalities at wind turbines. Obviously, this correlation is also lacking at pre-construction sites [43]. Another important observation is that sometimes, the variation in bat activity between nights, between years, and between different wind turbines might be high. There are suggestions on risky areas for bat collisions, e.g., along coastlines, and more safe areas, e.g., in open areas or some types of forests [31,45]. However, this study shows that there might be a large number of bats at nacelle-height also in ”safe areas”.

In total, our observations indicate that predictions on bat activity at wind-power farms are very difficult to make. It highlights the need for long-term (several months) surveys at nacelle level. Though bat behaviour might change when the wind turbines are erected [46,47,48,49], surveys at nacelle height are probably more correct than those made only at ground level.

Obviously, both the technical systems and the settings are important to be able to achieve recordings with both high sensitivity and low noise. In our case, D500X was more reliable (in terms of the number of nights when it worked), but it was possible to use higher sensitivity on Avisoft due to an efficient noise-reducing filter system, thereby enabling the detection of more bats. We conclude that there is a risk of misjudgements in environmental impact assessments depending on technical systems, and different systems might need different interpretations.

Our results are important in relation to mitigation measurements, and especially stop-regulation based on weather conditions. Rydell et al. [22] reviewed studies of bat occurrence at nacelle height compared to weather conditions in Sweden and found that 90% of all bat observations were registered when the temperature was >14 °C, and the wind speed <5.8 m/s. In the present study, within these limits, only 53% of the bats would be captured. According to our results, 90% of bat passes were was registered when the temperature was 9 °C, and the wind speed was <8.2 m/s. Our study is only based on measurements at three wind turbines for two years, and therefore not enough to draw general conclusions for stop-regulation. However, this study highlights some of the problems with setting the limits for stop-regulation. The variation in bat activity was high between different nights and wind turbines, and the bat activity was sometimes high, even in windy conditions. An alternative to stop-regulation only based on weather conditions would be to use stop-regulation based on both real-time bat activity and weather conditions [29].

## 5. Conclusions

Bats forage around wind turbines and based on this study, the feeding attraction hypothesis cannot be rejected. However, our correlation between insect abundance and bat activity was weak and the relation between bat activity and weather conditions were stronger. We cannot conclude that foraging is the only reason for visiting wind turbines at nacelle-height, and maybe not even the main reason. This means that insect-reducing measures, such as changes in light and colour, may have limited effect. Due to high variability in bat activity in space and time (including both low-risk and high-risk species), and difficulties in predicting bat activity, we argue that long-term surveys should be standard in environmental impact assessment, preferably at nacelle height.

## Figures and Tables

**Table 1:** Summary of the sampling results concerning the bat recording. The first and last night represent the first and last date respectively when the equipment worked. Number of nights represents the number of nights where the equipment worked. Number of recordings represent the total number of recording during the study period. Total bat activity represents the number of bat passes for the all study period. Bat activity per night is the mean (number of bat passes) ± SE per night. Note that the data from SWT, 2018, at the nacelle were not used for the analyses due to the low number of nights where the recorder worked.

Year	Height	Variable	SWT		NWT1	NWT2	Total
			Avisoft	D500X	Avisoft	Avisoft	
2017	Nacelle	First night	29 June	-	15 June	-	-
		Last night	29 October	-	31 October	-	-
		Number of nights	104	-	139	-	243
		Number of recordings	19943	-	6785	-	26728
		Total bat activity	2718	-	59	-	2777
		Bat activity per night	26.1 ± 7.3	-	0.4 ± 0.1	-	11.4 ± 3.2
	Ground	First night	28 June				-
		Last night	29 October				-
		Number of nights	91	-	-	-	91
		Number of recordings	15442	-	-	-	15442
		Total bat activity	262	-	-	-	262
		Bat activity per night	2.9 ± 0.9	-	-	-	2.9 ± 0.9
2018	Nacelle	First night	5 July	5 July	1 June	1 June	-
		Last night	30 July	21 October	31 October	31 October	-
		Number of nights	26	95	137	145	403
		Number of recordings	19	157764	456	830	159069
		Total bat activity	6	57	362	605	1 030
		Bat activity per night	0.2 ± 0.1	0.6 ± 0.2	2.6 ± 1.3	4.2 ± 1.2	2.5 ± 0.6
	Ground	First night	5 July	5 July	-	-	-
		Last night	31 October	31 October	-	-	-
		Number of nights	119	114	-	-	233
		Number of recordings	4000	30806	-	-	34806
		Total bat activity	2130	1090	-	-	3220
		Bat activity per night	17.9 ± 3.1	9.6 ± 1.3	-	-	13.8 ± 1.3

**Table 2:** The mean value and SE of the average wind speed per night and average temperature per night calculated for the whole survey period. Mean value and SE of the nightly precipitation on rainy nights (45 nights).

Site	Wind speed (m/s)		Temperature (°C)		Precipitation on Rainy Nights (mm)	
	2017	2018	2017	2018	2017	2018
SWT	7.3 ± 0.2	7.4 ± 0.2	11.8 ± 0.3	13.9 ± 0.5	3.1 ± 0.9	3.2 ± 1.4
NWT1	6.7 ± 0.2	6.8 ± 0.2	11.7 ± 0.3	14.4 ± 0.4		
NWT2	-	6.6 ± 0.2	-	14.5 ± 0.4		

**Table 3:** Results from the multiple Poisson regressions, where the total bat activity or the activity of the five most common taxa were used as the response variable. n = 87 nights (from SWT at nacelle height in 2017). Numbers in parenthesis are the Wald χ2 test statistic. * = *p* < 0.05, ** = *p* < 0.01, *** = *p* < 0.001, ns = not significant but included in the model with the lowest AIC, ni = not included in the model with the lowest AIC.

Variable	All Species	*Pipistrellus pygmaeus*	*Eptesicus nilssonii* ^1^	*Myotis spp.*	*Nyctalus noctula*	*Vespertilio murinus*
Wind	(19.85) ***	(17.24) ***	ni	(21.93) ***	(8.36) **	ni
Temperature	(56.14) ***	(84.34) ***	(77.73) ***	ni	ni	(8.61) **
Precipitation	ni	ni	ni	(2.10) ns	(3.21) ns	(2.86) ns
Insect abundance	(4.10) *	ni	(5.71) *	ni	(24.43) ***	(46.38) ***

^1^ The overdispersion of this model could not be compensated because of one night with 550 bat passes of *E. nilssonii*, therefore models with the activity of *E. nilssonii* as the response variable has been done by removing this night.

**Table 4:** Bat activity (number of bat passes) ± SE per night for the five most common species, recorded with Avisoft equipment, at NWT1 at nacelle-height (n = 137 nights) and at SWT at ground level (n = 91 nights) in 2017 and 2018. Results from the Wilcox test that compared the activity of each species between the two years.

Turbine (Height)	Species	2017	2018	Results from Wilcox test
NWT1 (nacelle-height)	*E. nilssonii*	0.14 ± 0.06	1.20 ± 0.69	W = 9230, *p* = 0.28
	*P. pygmaeus*	0.11 ± 0.07	0.45 ± 0.29	W = 9441, *p* = 0.72
	*Myotis* spp.	0	0	-
	*N. noctula*	0.13 ± 0.07	0.73 ± 0.36	W = 9156, *p* = 0.19
	*V. murinus*	0.04 ± 0.08	0.18 ± 0.07	W = 9167, *p* = 0.12
SWT (ground level)	*E. nilssonii*	1. 37 ± 0.48	2.84 ± 0.63	W = 3046.5, *p* < 0.01
	*P. pygmaeus*	0.62 ± 0.21	7.77 ± 2.72	W = 2504, *p* < 0.01
	*Myotis* spp.	0.54 ± 0.20	2.71 ± 0.73	W = 2583, *p* < 0.01
	*N. noctula*	0.15 ± 0.07	2.37 ± 0.69	W = 3303, *p* < 0.01
	*V. murinus*	0.15 ± 0.08	0.30 ± 0.09	W = 3907, *p* = 0.19

**Table 5:** Bat activity (number of bat passes) ± SE per night at the nacelle height for the five most common species, recorded with Avisoft equipment (n = 104 nights in 2017 and n = 137 nights in 2018). Results from the Wilcox test that compared the activity of each species between two wind turbines.

Year	Species	SWT	NWT1	NWT2	Results from Wilcox Test
2017	*E. nilssonii*	8.13 ± 5.3	0.12 ± 0.07		W = 76, *p* < 0.01
	*P. pygmaeus*	8.38 ± 1.8	0.14 ± 0.09		W = 87, *p* < 0.01
	*Myotis* spp.	7.95 ± 1.9	0		W = 90, *p* < 0.01
	*N. noctula*	0.45 ± 0.1	0.17 ± 0.10		W = 60, *p* < 0.05
	*V. murinus*	0.69 ± 0.2	0.06 ± 0.04		W = 68, *p* < 0.01
2018	*E. nilssonii*		1.20 ± 0.73	1.74 ± 0.76	W = 10, *p* < 0.05
	*P. pygmaeus*		0.45 ± 0.30	0.58 ± 0.38	W = 95, *p* = 0.59
	*Myotis* spp.		0	0	-
	*N. noctula*		0.73 ± 0.38	1.12 ± 0.41	W = 11, *p* < 0.01
	*V. murinus*		0.18 ± 0.08	0.57 ± 0.17	W = 10, *p* < 0.05

## Data Availability

Restrictions apply to the availability of these data. Part of the data was obtained from Erikhester Vindpark AB, and are available from Johnny de Jong (johnny.de.jong@slu.se) with the permission of Erikhester Vindpark AB.

## Appendix A

**Table A1:** Settings of Avisoft and the D500X.

Parameters	Avisoft SWT	Avisoft NWT	D500X SWT
Pre-trigger	0.5s	0.5s	OFF
Hold	2	4.5s	
Level	0,35	0.35	
Range	14–70 kHz	14–70 kHz	
Entropy	No	Y = 35%	
Reject wind/rain	No	No	
Whistle tracking	No	No	
Max file size	0.12 min = 7.2 s	0.09 min = 5.4 s	5 s
Interval between recording			5 s
Sample frequency			500
Trigger sens.			High
Input gain			60
Trig level			30
Bat call filter	Yes	Yes	
Bat call filter settings	Standard	Standard	
HP Filter			Yes
**Time programme**			
Type	absolute timer	absolute timer	absolute timer
From	19:00	19:00	19:00
To	06:00	06:00	06:00
